# Roles of G Protein-Coupled Receptors (GPCRs) in Gastrointestinal Cancers: Focus on Sphingosine 1-Shosphate Receptors, Angiotensin II Receptors, and Estrogen-Related GPCRs

**DOI:** 10.3390/cells10112988

**Published:** 2021-11-03

**Authors:** Zhen Zeng, Chunxiang Ma, Kexin Chen, Mingshan Jiang, Reshma Vasu, Rui Liu, Yinglan Zhao, Hu Zhang

**Affiliations:** 1 Department of Gastroenterology, West China Hospital, Sichuan University, Chengdu 410061, China; 2017224025220@stu.scu.edu.cn (Z.Z.); 2018224025234@stu.scu.edu.cn (C.M.); chenkexin1@stu.scu.edu.cn (K.C.); jmsalice@163.com (M.J.); 2 Centre for Inflammatory Bowel Disease, West China Hospital, Sichuan University, Chengdu 410061, China; 3 Lab of Inflammatory Bowel Disease, Frontiers Science Center for Disease-Related Molecular Network, West China Hospital, Sichuan University, Chengdu 410061, China; 4 West China School of Medicine, Sichuan University, Chengdu 410061, China; reshmavasu@yahoo.com; 5 Key Laboratory of Green Chemistry & Technology, College of Chemistry, Sichuan University, Chengdu 610064, China; liur@scu.edu.cn; 6 Cancer Center, West China Hospital, West China Medical School, Sichuan University, and Collaborative Innovation Center for Biotherapy, Chengdu 610041, China; zhaoyinglan@scu.edu.cn

**Keywords:** G protein-coupled receptors, colorectal cancer, gastric cancer, esophageal cancer, drug discovery

## Abstract

It is well established that gastrointestinal (GI) cancers are common and devastating diseases around the world. Despite the significant progress that has been made in the treatment of GI cancers, the mortality rates remain high, indicating a real need to explore the complex pathogenesis and develop more effective therapeutics for GI cancers. G protein-coupled receptors (GPCRs) are critical signaling molecules involved in various biological processes including cell growth, proliferation, and death, as well as immune responses and inflammation regulation. Substantial evidence has demonstrated crucial roles of GPCRs in the development of GI cancers, which provided an impetus for further research regarding the pathophysiological mechanisms and drug discovery of GI cancers. In this review, we mainly discuss the roles of sphingosine 1-phosphate receptors (S1PRs), angiotensin II receptors, estrogen-related GPCRs, and some other important GPCRs in the development of colorectal, gastric, and esophageal cancer, and explore the potential of GPCRs as therapeutic targets.

## 1. Introduction

Gastrointestinal (GI) cancers are common and devastating diseases with high global incidence and prevalence rates. Available data indicate that the number of new cases of GI cancers is 5.0 million, causing 3.5 million related deaths in 2020 [1]. Colorectal, gastric, liver, esophageal, and pancreatic cancer are the top five malignancies of the GI tract, accounting for 26.1% of the total cancer incidence and 35.6% of the cancer-related deaths in 2020 [1]. It is estimated that the global number of new cases and related deaths of GI cancers will substantially increase to 7.5 and 5.6 million by 2040, respectively [2,3]. Such high incidence and mortality rates pose a great challenge to public health, economic growth, and social development.

Despite the significant advances that have been made in the understanding of the pathogenesis of GI cancers, the exact pathogenesis has not yet been made clear. The available data suggest that GI cancers result from a complex interaction between environmental factors, microbiome, diet, and aberrant immune responses in genetically susceptible individuals [4]. Recent progress in exploring the structures, dynamics, and functions of G protein-coupled receptors (GPCRs) has provided new insights into the complicated pathogenesis of GI cancer. Given the important contributions of GPCRs in mediating cell proliferation, migration, and invasion, as well as angiogenesis, cell death, and cell survival, GPCRs play critical roles in cancer growth and development [5]. Significant associations between GPCRs and GI cancer have been established in several studies, indicating that GPCRs are appealing therapeutic targets for GI cancers.

In recent years, aberrant lipid metabolism was found to play a critical role in tumorigenesis and antitumoral therapy response [6,7]. Lipid metabolism significantly affects tumorigenesis by regulating cell growth, proliferation, and death. It is also associated with antitumoral therapy [8]. Various kinds of cancers present abnormal lipid catabolic and anabolic processes, making them become resistant to cancer therapy [6]. Moreover, the renin-angiotensin system (RAS) also received substantial interest in the field of cancer research. A close association between the RAS and tumorigenesis has been found in several studies [9,10,11]. As integral parts of the RAS, angiotensin II receptors, including angiotensin II receptor type 1 (AT1R) and angiotensin II receptor type 2 (AT2R) are implicated in tumor angiogenesis and tumor metastasis by modulating vascular wall thickness, vascular injury, and cytokine secretion [9]. As a result, the epithelial-to-mesenchymal transition (EMT) and tumor microenvironment (TME) are changed, interfering with cancer growth and progression [10,11]. Furthermore, GI cancers show sexual dimorphism in incidence and mortality, which has attracted considerable interest in evaluating roles of estrogen and estrogen-related GPCRs in the initiation and development of GI cancers including colorectal, gastric, and esophageal cancer [1,12,13,14]. These estrogen-related GPCRs and associated signaling pathways regulate TME and EMT, the characteristic features of cancers, therefore modulating cancer behavior and the antitumoral therapy response [15,16]. From this point, estrogen-related GPCRs are appealing therapeutic targets for GI cancer. Considering that lipid metabolism, angiogenesis, and estrogen were strongly associated with colorectal, gastric, and esophageal cancer, sphingosine 1-phosphate receptors (S1PRs), angiotensin II receptors, and estrogen-related GPCRs were extensively investigated in GI cancers.

In this review, we briefly introduce the structures and signaling pathways of GPCRs and discuss the roles of S1PRs, angiotensin II receptors, estrogen-related GPCRs, and some other important GPCRs in the development of colorectal, gastric, and esophageal cancer. Furthermore, we also evaluate the therapeutic potential of targeting GPCRs and the associated signaling pathways in the prevention and treatment of GI cancers.

## 2. Structures and Signaling Pathways of GPCRs

As the largest family of membrane receptors, GPCRs are involved in regulating diverse pathophysiological processes, thus attracting considerable interest in recent years [17,18]. GPCRs are characterized by the seven α-helical transmembrane domains, the extracellular domains (ECDs), and the intracellular domains (ICDs) [5]. Based on sequence and structural similarity, GPCRs can be divided into six families, termed A through F. The largest family, the class A family, comprises numerous rhodopsin-like receptors, therefore becoming the predominant targets in GPCR-based therapeutics [19]. Other classes, including the secretin receptor family (class B), the glutamate family (class C), the fungal mating pheromone receptor family (class D), the cyclic adenosine monophosphate (cAMP) family (class E), and the frizzled family (class F), also show great potential in drug discovery [20]. Recent data indicate that more than one third of the United States Food and Drug Administration (FDA)-approved drugs are GPCR-targeted drugs [21]. In 2019, GPCR-targeted small molecule drugs account for 22% of the new orally available small molecule drugs [22].

**Figure 1:**
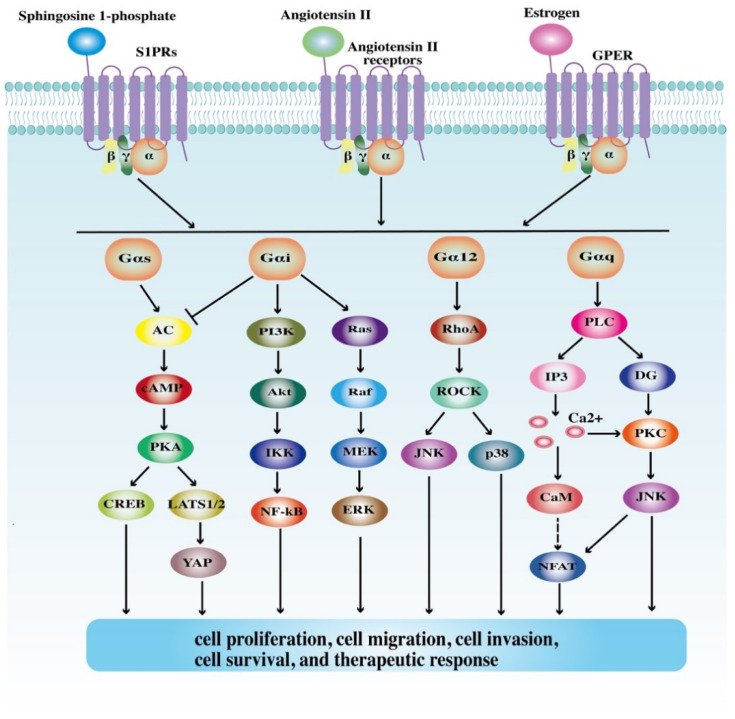
Roles of G protein-coupled receptors (GPCRs) in tumorigenesis and therapeutic response. Various ligands bind to sphingosine 1-phosphate receptors (S1PRs), angiotensin II receptors, and G protein-coupled estrogen receptor (GPER) and activate downstream pathways including adenylyl cyclase (AC)/cyclic adenosine monophosphate (cAMP)/protein kinase A (PKA), phosphatidylinositol 3 kinase (PI3K)/Akt, Ras/mitogen-activated extracellular signal-regulated kinase (MEK)/extracellular signal-regulated kinase (ERK), ras homolog family member A (RhoA)/Rho associated coiled-coil containing protein kinase (ROCK), phospholipase C (PLC)/inositol triphosphate (IP3), and PLC/diacylglycerol (DG)/protein kinase C (PKC) signaling pathways, regulating cell proliferation, migration, invasion, survival, and therapeutic response.

GPCRs can respond to a variety of ligands, ranging from small molecules to peptides and proteins. Odorants, neuropeptides, chemokines, etc., are important ligands for GPCRs [20]. Upon being bound with ligands, GPCRs can transform from inactive conformational states to active ones and subsequently activate G proteins, the heterotrimeric guanine-nucleotide-binding signal transducing proteins (Gs, Gi/o, Gq, and G12). As a result, multiple signaling cascades such as the adenylyl cyclase (AC)/cAMP, phospholipase Cβ (PLC-β)/Ca^2+^, RhoA/ Rho associated coiled-coil containing protein kinase (ROCK), Ras/extracellular signal-regulated kinase (ERK), phosphatidylinositol 3 kinase (PI3K)/Akt, and WNT/β-catenin pathways are activated. These signaling pathways then regulate various cancer-associated processes such as cell proliferation, migration, and invasion in a direct or indirect way [5,9,23] (Figure 1). Moreover, it should be noted that GPCRs can activate G proteins as monomers, and also as homodimers, heterodimers, and even high-order oligomers through dimerization with other receptors [24]. Dimer and oligomer GPCRs show great differences in receptor activation, desensitization, and internalization from monomer GPCRs, therefore mediating distinct biological processes from monomer GPCRs [25]. Furthermore, G protein-coupled receptor kinases (GRKs) are also implicated in GPCR signaling pathways. GRKs phosphorylate ICDs of GPCRs, leading to the recruitment of β-arrestins. This process results in receptor desensitization, internalization, and degradation, thereby interfering with downstream transduction pathways [26]. As signaling scaffolds, β-arrestins also regulate intracellular signaling networks in a G protein-independent fashion. They are involved in the activation of c-Jun N-terminal kinase (JNK), p38mitogen-activated protein kinase (MAPK), ERK, and nuclear factor kappa-B (NF-κB), thus mediating cell proliferation, migration, and invasion [27]. Collectively, the structures and signaling pathways of GPCR are quite complex. GPCRs are extensively involved in cancer-related cellular processes, highlighting the need to clarify the roles of GPCRs in the development of GI malignancies and explore more GPCR-based therapeutics for GI cancers.

## 3. Roles of GPCRs in GI Cancers

### 3.1. Colorectal Cancer

Colorectal cancer (CRC) is the most common GI cancer, with 1.9 million new cases and 0.9 million deaths in 2020, becoming the third leading cause of cancer and the second leading cause of cancer mortality in 2020 [1]. Although significant achievements have been made in the therapy of CRC, the mortality remains high [28]. Several lines of evidence indicate that the progression of CRC is a multi-factor and multi-step process [29]. As an important factor for the progression of CRC, GPCRs have provided new insights into CRC tumorigenesis. In this part, we mainly focus on the roles of sphingosine 1-phosphate receptors, angiotensin II receptors, estrogen-related GPCRs, and some other important GPCRs in CRC (Table 1).

#### 3.1.1. Sphingosine 1-Phosphate Receptors

In recent years, metabolic reprogramming was found to play a major role in tumorigenesis. A number of studies have linked lipid metabolism to cell growth, proliferation, and death as well as therapeutic success and resistance [6]. Phospholipids are a class of lipids with a backbone of sphingoid bases. Many metabolites of phospholipids such as sphingosine 1-phosphate (S1P) are involved in a variety of cellular processes including cell growth, proliferation, and migration, as well as angiogenesis, immune responses, and inflammation [50]. Sphingosine kinases (SphKs, including SphK1 and SphK2) phosphorylate phospholipids to S1P. S1P binds to and activates S1PRs (including S1PR1-5), class A family GPCRs, mediating the wingless-type family member 5A (Wnt5A)/β-catenin, epidermal growth factor receptor (EGFR)/PI3K/Akt, NF-κB/IL-6/ signal transducer and activator of transcription 3 (STAT3), and cyclooxygenase-2 (COX-2)/prostaglandin E2 (PGE2) signaling pathways [51].

In 2006, Kawamori et al. [52] proposed that the SphK1/S1P signaling pathway could accelerate CRC progression by increasing the production of COX-2 and PGE2. Subsequently, Kawamori and colleagues [53] firstly showed that the progression of CRC in SphK1^−/−^ mice was significantly blunted when compared with wild-type (WT) mice. They also found that the expression levels of SphK1 were higher in human colon cancer tissues than in normal colon mucosa. Most importantly, metastatic CRC tissues presented higher levels of SphK1 when compared with nonmetastatic CRC tissues [53]. This finding proved that the SphK1/S1P pathway could augment the metastatic potential of CRC. Meanwhile, Liang et al. [30] also found that both SphK1 and S1PR1 were upregulated in colitis-associated cancer (CAC), and S1P was implicated in CAC development through its activation of the NF-κB/IL-6/STAT3/S1PR1 amplification loop. Fingolimod, an agonist of four S1PRs (including S1PR1, S1PR3, S1PR4, and S1PR5), induces receptor internalization and exerts antagonistic effects, which could markedly reduce the tumor load and abrogate the NF-κB/IL-6/STAT3/S1PR1 cascade [30]. From this point, SphK/S1P/S1PR and associated signaling pathways are of great importance in the development of CRC.

As for the roles of S1PR2 in the development of CRC, Petti et al. [31] claimed that S1PR2 suppressed CRC and CAC progression by preventing epithelial stem cell proliferation and blocking their malignant transformation. Overexpression of S1PR2 increased the levels of phosphatase and tensin homolog deleted on chromosome ten (PTEN) and axis inhibition protein 2 (Axin2), and then down-regulated the PI3K/Akt and Wnt/β-catenin signaling pathway, respectively. As a result, cell proliferation and malignant transformation are suppressed [31]. Similarly, S1PR2 also showed anti-tumor effects on B-cell lymphoma and melanoma by inhibiting tumor growth, invasion, and migration [54]. It is noteworthy that the role of S1PR2 in tumorigenesis is controversial. Several other studies proposed that S1PR2 promoted the development of Wilms tumor, liver cancer, glioma by activating c-Jun, c-Fos, COX-2, and ERK/MAPK [55,56,57]. In addition, studies also linked S1PR2 to drug resistance. Zhang et al. [32] found that a specific inhibitor of S1PR2 (JTE-013) markedly reduced the expression of dihydropyridine dehydrogenase (DPD) and reversed 5-FU resistance in vivo and in vitro. This result represented a promising field of sensitizing 5-FU therapy in CRC. S1PR5 also shows bidirectional roles in cancer behavior. In CRC cell lines, the overexpression of S1PR5 resulted in an increased expression of phospho-p65, thus activating the NF-κB/indoleamine 2,3 dioxygenase 1 (IDO1) signaling pathway and promoting colon cancer cell proliferation and invasion [35]. Nevertheless, S1PR5 may negatively regulate cell proliferation and migration in esophageal squamous cell carcinoma cell lines through the Ras/ERK, PI3K/Akt, and Rho/ROCK signaling pathways [58]. Regarding two other S1PRs (S1PR3 and S1PR4), the available evidence demonstrated pro-tumor effects in various cancers including CRC, gastric cancer, and breast cancer [33,34,54]. S1PR3 was claimed to be involved in the regulation of CRC cell proliferation, migration, invasion, and apoptosis by modulating the Akt and ERK pathways [33]. S1PR4 plays a tumor-promoting role in CRC. Notably, S1PR4 also hindered chemotherapy by reducing the expansion of antitumor CD8+ T cell. These pro-tumor effects were also confirmed in breast cancer cell lines [34].

Given that some S1PRs show bidirectional effects on tumorigenesis, underlying mechanisms such as different TME, different affinities of S1PRs, and diverse signaling pathways should be further clarified. Furthermore, gut microbiota plays a key role in CRC and GI inflammation, and therefore, is in an area of active investigation [59,60]. However, little information is available on the roles of the SphK/S1P/S1PR signal in microbial pathogenesis [4]. Exploring the interaction between the SphK/S1P/S1PR signal and microbial communities may have broad significance toward understanding the initiation and development of CRC (such as the process from GI inflammation to CAC).

#### 3.1.2. Angiotensin II Receptors

AT1R and AT2R are the two main members of angiotensin II receptors, belonging to class A GPCRs. AT1R shares 30% of amino acid sequence homology with AT2R and shows some opposite effects from AT2R [9,61]. Renin, angiotensin II, AT1R, AT2R, and angiotensin converting enzyme (ACE) are integral parts of the RAS. After being stimulated by angiotensin II, AT1R activates Gq and G12 and subsequently triggers the PLC-β/Ca^2+^ and RhoA/ROCK signaling cascades [61]. A variety of downstream transduction pathways including Ras/ERK, PI3K/Akt, and Janus kinase (JAK)/STAT have also been identified [9]. AT1R is implicated in tumor angiogenesis and TME modulation by regulating vascular wall thickness, vascular injury, and cytokine secretion, thus playing a vital role in cancer progression [9,10]. On the contrary, AT2R exerts anti-tumor effects by promoting apoptosis, inhibiting inflammation, and suppressing the MAPK and ERK signaling pathways [9,62].

A large case–control study of 2,847 cases and 28,239 controls suggested that long-term use of angiotensin converting enzyme inhibitors (ACEIs)/angiotensin receptor blockers (ARBs) was associated with a decreased risk of CRC in patients with hypertension [63]. In the same year, another population-based cohort study of 297,688 subjects also demonstrated protective effects of ACEIs and ARBs on the development of cancer [64]. Recently, a meta-analysis of 16 studies (2,847,597 patients) claimed that the CRC risk and overall mortality were reduced in patients treated with ACEIs/ARBs in comparison to those without ACEI/ARB treatment [65]. There remain controversies over the effects of ACEIs/ARBs on cancer risk. Several other studies drew contrasting conclusions that the use of ACEIs/ARBs was irrelevant to cancer risk [66,67,68]. Different research designs and cancer types, as well as an inherent publication bias may account for these inconsistent findings. Even so, in many clinical trials, cancer patients were treated with ACEIs/ARBs and benefited from ACEIs/ARBs in terms of therapeutic efficacy and disease recurrence [69]. Based on this, some researchers further revealed the underlying mechanisms of ACEIs/ARBs in cancer.

An Australian research team investigated the effects of AT1R antagonist (irbesartan) and ACEI (captopril) on a mouse model of CRC liver metastases. Both irbesartan and captopril markedly decreased tumor growth and microvascular density, suggesting a great potential of developing drugs targeting AT1R and ACE [36]. Nguyen et al. [11] further provided important clues about the pro-tumor mechanisms of AT1R. They treated CRC cell lines with angiotensin II, AT1R blocker, and AT2R blocker. As a result, angiotensin II-mediated CRC cell migration was markedly reversed by the AT1R and AT2R blocker. Furthermore, blocking of AT1R repressed EMT, a biological process of converting epithelial cells to other cells with mesenchymal phenotypes [11,70]. In contrast, the AT2R inhibitor decreased the expression of E-cadherin, with no influence on ZEB1 or vimentin (mesenchymal markers) expression [11]. From this point, AT1R may exert pro-tumor effects by promoting EMT.

In addition, AT1R is also involved in the regulation of cancer-associated fibroblasts (CAFs), a type of functional cell modulating tumor stroma, cell proliferation, and therapeutic resistance [9,37]. The AngII/AT1R signal induced CAFs to produce transforming growth factor-β (TGFβ), thus increasing the expression of extracellular matrix proteins and remodeling the mechanical stress of tumor stroma, and then affecting the infiltration of drugs into the tumor [9]. Most importantly, ARBs were claimed to be critical contributors to the generation of an immunosupportive TME. Chauhan et al. [37] showed that ARBs reduced CAF activity, interfered with TGF-β and hypoxia signaling, decreased immunosuppressive CXCL13 production, and increased M1-like (anti-tumor) macrophage population. All these findings supported the critical roles of ARBs in the generation of an immunosupportive TME. In accordance with this, Nakamura and colleagues [38] also proposed that ARBs contribute to the generation of an immunosupportive TME in a mouse model of CRC. Additionally, then, ARBs boosted immunotherapy (anti-programmed death-ligand 1, anti-PD-L1) efficacy and repressed cancer progression [37,38]. Obviously, RAS plays a critical role in the regulation of TME.

Moreover, cancer stem cells (CSCs), a group of cancer cells with self-renewal characteristics and tumorigenic abilities, mediate tumor progression and therapeutic resistance [71,72]. Gao et al. [73] found that migrating cancer stem cells (MCSCs) were expressed in CRC tissues and CRC cell lines. Most importantly, they identified CD110^+^ and CDCP1^+^ MCSCs as candidate markers of organ-specific metastasis. In line with this finding, other studies further suggested that AT1R and AT2R were expressed on two subpopulations of CSCs (SOX2^+^ and OCT4^+^) in tissues of colon adenocarcinoma metastasis to the liver (CAML) [9,74]. In this regard, CSCs with different surface markers may be valuable in the prediction and assessment of cancer metastasis. However, little is known about the differences between CSCs with AT1R/AT2R expression and those without expression in terms of metastatic potential. Additional efforts are needed to fill this gap.

#### 3.1.3. Estrogen-Related GPCRs

It is well documented that CRC exhibits sexual dimorphism in incidence, mortality, and tumor type [12]. The age-standardized incidence rates of CRC are lower in women than in men, and young females with CRC display a lower mortality in comparison to age-matched males with CRC [1,12]. Additionally, multiple lines of evidence also demonstrated that hormone replacement therapy (HRT) and oral contraceptive use are associated with a lower risk of CRC [75,76,77]. In accordance with these findings, women with hysterectomy or oophorectomy were more prone to develop CRC compared with controls [78]. These results indicated a protective role of estrogen in CRC development. However, negative results have been obtained in some other studies. In these studies, greater endogenous estrogen exposure was claimed to be a contributor to CRC development [79]. Although results are inconclusive, estrogen indeed plays a role in CRC development. Estrogen is implicated in tumorigenesis through the engagement of different receptors including classic estrogen receptor α (ERα), estrogen receptor β (ERβ), and novel G protein-coupled estrogen receptor (GPER, also known as GPR30). Among these receptors, GPER and ERβ have received substantial interest in the field of cancer research.

Santolla et al. [39] firstly demonstrated that GPER was implicated in the modulation of fatty acid synthase (FASN) in cancer cells and CAFs, thus regulating neoplastic transformation of the colon through the EGFR/ERK/c-Fos/activating protein1 (AP1) signaling pathway. Other studies further revealed molecular pathways in CRC. Liu et al. [15] established that GPER suppressed the proliferative potential of CRC and blocked cell cycle progression, as well as accelerated mitochondrial-related apoptosis and endoplasmic reticulum (ER) stress by repressing the NF-κB signaling and activating the reactive oxygen species (ROS)/ERK signaling pathway. However, in another study, GPER was reported to promote CRC progression by increasing connective tissue growth factor [40]. GPER also increased the expression levels of hypoxia inducible factor (HIF) and vascular endothelial growth factor (VEGF) under hypoxic TME, thereby promoting cancer invasion and metastasis [41]. Meanwhile, contrary results have also been obtained in clinical research studies. Some researchers claimed a protective effect of GPER on CRC progression, in view of the fact that patients with a higher expression of GPER had a higher survival rate as compared with cases with lower expression levels [15,80]. However, others claimed a detrimental role of GPER in CRC and cited the association of increased GPER with poorer relapse-free survival [12,41]. Although the conclusions are inconclusive, GPER certainly contributes to colorectal carcinogenesis. Interpreting these results proved to be extremely difficult due to various factors such as age distribution, regional distribution, sexual dimorphism, clinical stage, and ethnicity, which significantly affected final conclusions. Furthermore, the use of in vivo and in vitro studies, different CRC cell lines, and different mouse models (azoxymethane/dextran sulfate sodium, AOM/DSS induced CAC model or xenograft CRC model) also interfered with interpretation of the data.

It is noteworthy that a close association between ERβ and gut microbiota has been confirmed in CRC studies [81]. Ibrahim et al. [81] provided preliminary evidence that a loss of intestinal epithelial ERβ decreased gut microbiota diversity in AOM/DSS-induced mouse models. Moreover, ERβ was also involved in the regulation of epithelial barrier integrity, carbohydrate metabolism, and cell motility by affecting specific microbiota species [81]. Therefore, ERβ was claimed to be beneficial in attenuating CAC development. However, studies into the interplay between gut microbiota and GPER are still lacking. Further investigations are needed to address this aspect. Moreover, it should be emphasized that CAC and sporadic CRC show great differences in tumorigenesis. Hence, conducting comparative studies and unveiling different roles of estrogen-related GPCRs in them have become a critical step in understanding the complex pathogenesis of CRC. This also holds the key to precision treatment in CRC.

#### 3.1.4. Other GPCRs

Short-chain fatty acids (SCFAs) are the most abundant metabolites produced by gut microbiota in the colon [42]. They could activate free fatty acid receptors (FFARs) including FFAR2 (GPR43), FFAR3 (GPR41), and GPR109A to regulate cancer progression [42,82]. With regard to the roles of FFAR2 in malignant transformation, conclusions remain controversial. Some claimed a destructive role of FFAR2 in CRC development due to the fact that the FFAR2 level was markedly increased in CRC specimens compared with paired noncancerous tissues [43]. While others drew a contrasting conclusion that FFAR2 suppressed colon tumorigenesis [42,44,83], Tang et al. [44] demonstrated that FFAR2 exerted inhibitory effects on CRC proliferation by inducing cell-cycle arrest in G0-phase and activating caspases. Recently, another research team further unveiled the underlying mechanism of FFAR2 in vivo and in vitro. Lavoie et al. [42] proposed that loss of FFAR2 potentiated colon tumorigenesis by breaking the epithelial barrier, accelerating CD8^+^ T-cell exhaustion, and increasing IL27^+^ dendritic cells (DCs). Therefore, FFAR2 exerted anti-tumor effects on CRC development. Other FFARs such as GPR109A and FFAR3 are also implicated in tumorigenesis. GPR109A was involved in the differentiation of anti-inflammatory T-regulatory (Treg) cells and IL-10-producing T cells, thus protecting the colon against inflammation and tumorigenesis [45]. FFAR3 was claimed to be a possible tumor promoter in the colon, considering that it enhanced cell proliferation and inhibited apoptosis by interfering with histone acetylation [45]. Other GPCRs including cholecystokinin 2 receptor (CCK2R) and GPR56 have also become interesting targets of CRC research. CCK2R promoted colon carcinogenesis via increasing colonic progenitor cells and repressing bone morphogenetic protein 2 (BMP2) [46,47]. GPR56 enhanced EMT by activating the PI3K/AKT signaling and promoted chemoresistance through the RhoA-mediated pathway [48,49]. Taken together, exploring roles of GPCRs not only cast light on the complicated pathogenesis of CRC, but also paved a new way to CRC treatment. It should be noted that studies on the molecular mechanisms of CRC are still lacking. Therefore, more efforts are needed to further elucidate the underlying mechanisms and explore new therapeutic targets of CRC.

### 3.2. Gastric Cancer

With 1,089,103 new cases and 768,793 deaths in 2020, gastric cancer is the fifth most common cause of cancer morbidity and the fourth most common cause of cancer-related death globally [1]. It is well established that genetic susceptibility, *Helicobacter pylori* (*H pylori*) infection, and unhealthy lifestyles are risk factors in the development of gastric cancer [84]. Despite the remarkable achievements that have been made in the field of *H pylori* eradication therapy, endoscopic treatment, and checkpoint inhibition, the median survival is less than one year in advanced gastric cancer [85]. Exploring new therapeutic targets for gastric cancer is a pressing need. GPCRs are extensively involved in cancer-related cellular processes including cell proliferation, invasion, and migration (Table 2). Therefore, the roles of GPCRs in the initiation and development of gastric cancer merit further investigation.

#### 3.2.1. Sphingosine 1-Phosphate Receptors

As aforementioned, S1PRs are appealing targets in the treatment of GI cancers. In a previous study, Yamashita et al. [87] analyzed the expression profiles of S1PRs in human gastric cancer cell lines. They found that S1PRs were variably expressed in different gastric cancer cell lines. S1P showed pleiotropic effects on cancer cell migration. When S1P bound to and activated S1PR2, the migration of gastric cancer cells (AZ-521, exclusively expressed S1P2) was blunted. While bound to S1PR3, S1P promoted gastric cancer cell (MKN1 and HCG-27, predominantly expressed S1PR3) migration [87]. From this point, the anti-tumor/pro-tumor effects of S1P in gastric cancer partly depend on the predominance of different types of S1PRs. In accordance with the findings of CRC, the SphK/S1P/S1PR signaling pathway is of great significance in gastric cancer development. Cell proliferation and invasion were inhibited when S1PR1 was repressed [86]. However, inconsistent with previous findings of S1PR2, the S1P/S1PR2 signaling induced tyrosine phosphorylation of c-Met, EGFR, and ERK. As a result, cell growth, migration, and invasion were accelerated [87,88,105]. These conflicting results merit further study. Likewise, EGFR interactions between S1P and HER2 have also been investigated. The available evidence indicated that the transactivation of HER2 (known as ErbB2) by S1P could promote cancer progression through subsequently activating the PI3K/Akt and Ras/MEK signaling pathways. This process is dependent on EGFR [106,107]. As for other growth factors, such as platelet derived growth factor (PDGF), studies primarily focused on ovarian cancer, chondrosarcoma, and glioblastoma [108,109,110]. S1P transactivated the PDGF receptor β (PDGFRβ) by binding to S1PR3, and subsequently triggered the PI3K/Akt and Ras/ERK transduction pathway [108]. Additionally, S1PR1 and PDGFRβ could also form a signaling complex, implicated in angiogenesis, cell invasion, and cell migration [107]. Therefore, further studies are warranted to elaborate on interactions between the S1P/S1PR axis and the PDGF/ PDGFR signal in gastric cancer and other types of cancer. Furthermore, limited information is available on the roles of S1PR4 and S1PR5 in gastric cancer, indicating the need to further reveal it.

Additionally, many studies also focused on roles of SphK1 in gastric cancer. The available evidence demonstrated that SphK1 protein expression levels were increased in human gastric cancer tissues, in comparison to the surrounding noncancerous specimens [111]. Most importantly, SphK1 can serve as a promising marker of cancer prognosis. The overall survival time of patients with a higher SphK1 expression was shorter when compared with those with a lower SphK1 expression [111]. Associations between SphK1 and the clinical stage of gastric cancer have also been confirmed in several other studies [4]. Notably, epigenetic modifications also shape the expression of SphK1. miR-124 down-regulated SphK1 by binding to 3′untranslated regions (3′UTRs) of *SPHK1* and subsequently suppressed the tumorigenicity of gastric cancer cells [112]. Other epigenetic alterations, including histone modification and long non-coding RNAs (LncRNAs), also regulated *SPHK1* at both the transcriptional and post-transcriptional levels [51,113]. More studies should be conducted to further elaborate on the functional roles of epigenetic modifications in SphK1.

Some studies have found the ‘crisscross transactivation’ in breast cancer cells [107]. The interplay between the SphK1/S1P/S1PR signal and estrogen-related receptors was implicated in cancer invasion and chemoresistance [114]. This paved a new way for further identifying the interactions in gastric cancer, because estrogen-related receptors and associated signaling pathways both play a key role in gastric cancer [115]. Furthermore, given the pivotal roles of *H pylori* infection and the SphK/S1P/S1PR signal in the progression of gastric cancer, studies into the interplay between *H pylori* and the SphK/S1P/S1PR signal are also required. This may have a profound impact on understanding the pathogenesis of gastric cancer and the development of new targets for cancer treatment.

#### 3.2.2. Angiotensin II Receptors

RAS components such as AT1R, AT2R, and ACE are expressed in gastric cancer cell lines and tissues. Compared with healthy controls, the expression levels of AT1R, AT2R, and the activity of ACE were increased [90]. Carl-McGrath et al. [89] analyzed local expression of RAS components in 45 patients with gastric cancer. The results suggested that both AT1R and AT2R were expressed in 72.4% of primary tumors and in 60.0% of lymph node metastases. Moreover, in the N87 and MKN45 gastric cancer cell lines, the addition of AT1R and AT2R inhibitors markedly suppressed the invasive ability, suggesting a promising role of angiotensin II receptors in gastric cancer metastasis [89]. In line with this, losartan (an AT1R antagonist) significantly inhibited the tumor size and tumor weight in a mouse model of gastric cancer [90]. It should be noted that *H pylori* infection, a definite contributor to gastric cancer, significantly increased the expression of AT1R and AT2R by three to four times than those without *H pylori* infection [116]. Sugimoto et al. [91] investigated the changes of AT1R and AT2R mRNA concentrations in gastric mucosa during *H pylori* infection. Surprisingly, they found that the mRNA levels of AT1R and AT2R gradually increased during *H pylori* infection, in parallel with the degree of inflammatory cell infiltration. This result indicated an integral role of angiotensin II receptors in gastric oncogenesis, given that *H pylori*-mediated inflammatory cell infiltration is a key cause of gastric cancer.

Other molecular mechanisms have also been explored in various studies. Huang et al. [92] observed that AT1R antagonist (TCV-116) down-regulated the expression of VEGF and reduced microvascular density, thus repressing angiogenesis and gastric cancer progression. In addition, AT1R antagonist also prevented gastric cancer progression by blocking the angiotensin II-induced overexpression of matrix metallopeptidase-2 (MMP-2) and MMP-9, critical components mediating tumor migration and invasion [93]. Similarly, a Japanese research team demonstrated that the Angiotensin II/AT1R signal could activate the ERK and NF-κB signaling pathways and significantly increase the expression of survivin (anti-apoptotic protein), thereby exerting pro-tumor effects. Candesartan, an AT1R antagonist, reversed the angiotensin II-mediated pro-tumor effects [94]. In recent years, angiotensin II receptors have attracted considerable attention in the field of gastric cancer, due to their involvement in the regulation of EMT. Okazaki et al. [95] provided evidence that candesartan could reduce the expression levels of TGF-β1 and α-smooth muscle actin (α-SMA) and elevate the concentrations of E-cadherin in gastric cancer, suppressing fibrosis and EMT. As a result, cell proliferation and metastatic spread were suppressed.

Contrary to AT1R, AT2R counteracts AT1R-mediated fibrosis and shows anti-fibrotic effects in several diseases such as pulmonary fibrosis and renal fibrosis [117]. A phase II clinical trial is currently evaluating the efficacy of compound 21 (an AT2R agonist) in treating pulmonary fibrosis (NCT02503657). However, little is known about the anti-fibrotic roles of AT2R in gastric cancer, especially in signet-ring cell carcinoma (SRCC). Since AT2R shows ligand-independent constitutive activity, analyzing ligand-induced changes of AT2R expression has proven to be difficult [118]. Moreover, there are very few specific AT2R agonists or antagonists, which create additional obstacles to further explore the structures, functions, and associated signaling pathways of AT2R [117]. Hence, further studies are needed to design more AT2R agonists and antagonists with high specificity.

#### 3.2.3. Estrogen-Related GPCRs

In line with CRC, a clear sexual dimorphism of gastric cancer has been observed around the world [1,13]. According to data from the GLOBAL CANCER OBSERVATORY, females exhibited lower incidence and mortality rates of gastric cancer in comparison with males [1]. HRT and oral contraceptive use have been reported to decrease the risk of gastric cancer [119]. Clinical evidence also indicated a protective role of estrogen in gastric cancer. Notwithstanding, it should be stressed that estrogen can bind and activate different receptors and causes varying biological processes. Studies on ERα and ERβ are abundant, but research on the roles of GPER in the development of gastric cancer remains limited.

Available data show that mRNA and protein levels of GPER were decreased in gastric cancer tissues and cell lines, as compared to normal tissues [96,120]. In addition, lower mRNA levels of GPER also indicated a poorer overall survival and disease-free survival, suggesting that GPER has anti-tumor effects on gastric cancer [120]. Tian et al. [120] analyzed co-expressed genes with GPER in The Cancer Genome Atlas Stomach Adenocarcinoma (TCGA-STAD) and found a significant association between EMT and these co-expressed genes. Therefore, they claimed that GPER inhibited tumorigenesis via regulating EMT [120]. Lee et al. [96] made a similar conclusion that the activation of GPER conferred tumor suppressive activity by stimulating ER stress-related apoptosis. However, another study showed contradictory results that GPER aggravated gastric cancer progression and metastasis by inducing PI3K/Akt-mediated EMT [97]. Moreover, Wang et al. [16] further explored its role in chemoresistance. The results suggested that GPER contributed to cisplatin (a key chemotherapeutic agent) resistance by enhancing EMT. From this point, GPER may be a novel therapeutic target in gastric cancer. Recently, a striking finding from a Japanese study suggested that GPER-expressing gastric chief cells did not contribute to gastric metaplasia and dysplasia [121]. Hata et al. [121] proposed that GPER-expressing gastric chief cells were eliminated without converting to progenitor cells during the development of metaplasia. This is contrary to the existing theory that gastric chief cells are the cellular origin of gastric cancer [122,123]. Whether GPER affects the dedifferentiation or transdifferentiation potential of gastric chief cells remains to be determined.

As for these two classic receptors (ERα and ERβ), some studies suggested ERα as a tumor suppressor given that ERα inhibited cell growth and proliferation, induced cell cycle arrest, and enhanced cell apoptosis [124]. Nevertheless, ERα was also claimed to accelerate gastric cancer progression by activating the c-Src/EGFR and PI3K/Akt signaling pathways [125,126]. Compared with ERα, the expression status of ERβ is more dominant in gastric cancer tissues and cell lines [13]. Many studies showed that overexpression of ERβ was associated with an early cancer stage and increased survival time [115,127]. Breast cancer patients with Tamoxifen exposure were more prone to gastric adenocarcinoma than non-users. Additionally, the latency between the two cancers was shorter in Tamoxifen users [128]. The risk SNPs of *ESR2* (ERβ gene, rs1271572, rs3020443, and rs2978381) have also been demonstrated to be correlated with overall survival in patients with gastric cancer [129]. However, a recent study proposed that ERβ promoted the development and progression of gastric cancer. Knockdown of the ERβ expression enhanced apoptosis and the autophagy of gastric cancer cells in a MAPK-mediated way, thus repressing cell proliferation and invasion [130].

The functional roles of GPER in gastric cancer are incompletely understood; therefore, further research is required to gain a better understanding of the molecular drivers of gastric cancer. Moreover, significant associations between peptic ulcers and estrogen-related GPCRs have been established in several studies [131,132]. However, the pathophysiology of how these GPCRs contribute to the progression from peptic ulcers to gastric cancer remains unclear. Furthermore, a detailed understanding of how GPER interplay with *H pylori* is still lacking. Further investigations are required to uncover these issues.

#### 3.2.4. Other GPCRs

C-X-C chemokine receptors (CXCRs) including CXCR1-7 play a critical role in tumorigenesis. Numerous studies are in progress to identify their roles in the development and progression of gastric cancer. Li et al. [98] claimed that overexpression of CXCR1 and CXCR2 was correlated with an advanced clinical stage. CXCR1 and CXCR2 promoted gastric cancer cell migration and invasion through the JNK-mediated and ERK-mediated signaling pathways [98]. However, a high level of CXCR3 was found to be associated with better overall survival [133]. Recently, CXCR3 was demonstrated to be implicated in regulating the immunotherapy response. Zhao et al. [99] suggested that M1-like tumor-associated macrophages (TAMs) improved the therapeutic efficacy of PD-L1/programmed cell death protein 1 (PD-1) in gastric cancer via the C-X-C chemokine ligand (CXCL) 9,10,11/CXCR3 signaling pathway. With regard to CXCR4, the stromal cell-derived factor-1 (SDF-1)/CXCR4 signal contributed to 5-FU chemosensitivity via modulating autophagy [101]. The CXCL12/CXCR4 axis was claimed to promote macrophage polarization toward M2-like phenotypes, and thus, enhance gastric cancer metastasis [100]. Several other studies also proposed that the CXCL16/CXCR6 and the CXCL12/CXCR7 axes promoted the proliferation and migration of gastric cancer cell [102,103]. Collectively, CXCRs were actively involved in cell proliferation, migration, and invasion, suggesting potential therapeutic targets for gastric cancer treatment and promising markers for gastric cancer monitoring. Given that gastrin is in close association with gastric cancer, CCK2R, the identified receptor for gastrin, became one of the major research interests [134,135]. Chang et al. [104] provided evidence that the lack of gastrin promoted CCK2R^+^ stem cell (Notch1^low^/Numb^+^) proliferation and increased symmetric stem cell division, leading to large mutational burden during gastric antral tumorigenesis. However, Lgr5^+^ stem cells (Notch1^high^), the cellular origin of antral tumors, were not affected by gastrin and tightly regulated by the Notch signaling pathway [104]. Until recently, little has been known about the possible interactions between CCK2R^+^ stem cell and Lgr5^+^ stem cells. More studies are warranted to further explain the difference of cell behavior between them.

### 3.3. Esophageal Cancer

Esophageal cancer is the eighth most common cancer and sixth most common cause of cancer-related death around the world, with 604,100 new cases and 544,076 deaths in 2020 [1]. Esophageal squamous cell carcinoma (ESCC) and esophageal adenocarcinoma (EAC) are the two main forms of esophageal cancer, exhibiting a different prevalence between the east and west. Asian and African patients are more prone to ESCC, while EAC is more prevalent in North American and Western European populations [136]. Esophageal cancer is a highly progressive malignancy. Approximately half of newly diagnosed subjects suffered from metastatic esophageal cancer and the five-year survival rate of those patients was less than 5% [137,138]. Therefore, identifying and treating patients at an early stage is of great clinical significance. It has become increasingly apparent that GPCR and related signaling pathways play a crucial role in the onset and progression of esophageal cancer. The current therapeutic options for esophageal cancer are far from desirable; therefore, elucidating roles of GPCRs in esophageal cancer may open new possibilities of developing novel and more effective therapeutics. In this section, we mainly focus on the roles of S1PRs, angiotensin II receptors, estrogen-related GPCRs, and some other important GPCRs in esophageal cancer (Table 3).

#### 3.3.1. Sphingosine 1-Phosphate Receptors

Overexpression of SphK1 is closely associated with the invasion and metastasis of esophageal carcinoma. Human ESCC tissues showed higher expression levels of SphK1 in comparison to adjacent normal tissues [154]. Available data also suggested that increased SphK1 markedly contributed to deep invasion, lymph node metastasis, and poor five-year overall survival, highlighting the exciting potential of SphK1 as a prognostic marker [154,155]. SphK1 may enhance cell invasion and metastasis in esophageal carcinoma by regulating the phosphorylation of EGFR [154]. It is well established that Barrett’s esophagus (BE) is the precancerous lesion of esophageal cancer. As a critical factor contributing to the development of BE and esophageal cancer, bile acids directly damage esophageal epithelial cells and mediate oxidative stress, DNA damage, COX-2 expression, and apoptosis, thus promoting cancer invasion [140,156,157]. Liu et al. [140] claimed that S1PR2 mediated the pro-tumor effects of taurocholate (a conjugated bile acid) in invasive EAC cells. Both taurocholate and S1P activated S1PR2 and stimulated the yes-associated protein (YAP) and β-catenin signaling pathways, thereby promoting cell invasion [140]. Moreover, studies also linked S1PR2 to EMT [4]. A large body of evidence proved that EMT is significantly correlated with poor disease outcomes such as local invasion and lymph node metastasis [158,159]. Existing data showed that taurocholate could promote TGF-β-induced EMT in an S1PR2-dependent mechanism [140]. Inhibition of S1PR2 by JTE-013 caused an increase in E-cadherin and a decrease in vimentin in EAC cells, thus repressing TGF-β-induced EMT [140]. In accordance with this, another study also confirmed the pro-tumor role of S1PR2 in esophageal cancer. Miller et al. [141] used a specific siRNA to down-regulate S1PR2 and found S1P-induced and TGF-β-induced activation of ERK1/2 was inhibited. Consequently, the migration and invasion of EAC cells were suppressed.

Regarding other S1PRs, in vivo and in vitro studies revealed that overexpression of S1PR1 may be a valuable marker for poor prognosis in patients with ESCC [139]. Moreover, knockdown of S1PR1 repressed the proliferation and enhanced the apoptosis of ESCC cells, indicating a pro-tumor effect of S1PR1 in ESCC [139]. S1PR1 regulated cell proliferation and apoptosis via increasing the phosphorylation of STAT3 and promoting the transcription downstream target genes [139]. As for S1PR3, Shi et al. [142] observed increased mRNA levels of S1PR3 in ESCC cell lines. Meanwhile, they also demonstrated that up-regulation of SIPR3 contributed to radiation-induced Akt phosphorylation and the therapeutic sensitivity of PI3Kα inhibitor [142]. Contrary to the pro-tumor roles of S1PR1, S1PR2, and S1PR3, S1PR5 was claimed to exert inhibitory effects on the proliferation and migration of esophageal cancer via the Ras/ERK, PI3K/Rac and Rho/ROCK signaling pathways. Importantly, down-regulation of S1PR5 may be an important escape mechanism for esophageal cancer [58].

Altogether, S1PR-mediated signaling pathways are crucial contributors to tumorigenesis. However, little research is focused on the roles of S1PR4 in the development of esophageal cancer. Thus, more studies are warranted to further reveal the roles of these S1PRs in esophageal carcinoma. Moreover, several studies demonstrated that the interaction between epigenetics and SphK1 modified cancer initiation and progression [51]. Its efficacy in esophageal cancer remains to be seen; therefore, more efforts should be undertaken to fill this gap.

#### 3.3.2. Angiotensin II Receptors

A meta-analysis of ten studies demonstrated that ACEIs/ARBs protected high-risk individuals from developing ESCC. The risk of ESCC in cases without ACEI/ARB treatment is 1.72 times higher than that in cases treated with ACEIs/ARBs [160]. In addition, Chen et al. [143] further analyzed the contributions of ACEIs/ARBs on ESCC progression. They compared clinical outcomes between ESCC patients with ACEI/ARB treatment and those without ACEI/ARB treatment and linked ACEI/ARB treatment to better overall survival. These clinical findings indicated a protective role of ACEIs/ARBs in ESCC.

Several studies further analyzed the underlying mechanisms. Chen et al. [143] provided evidence that ACEIs/ARBs effectively suppressed cell proliferation and VEGF secretion in vitro. As it known to all, VEGF is a strong angiogenesis factor, implicated in neovascularization and tumor metastasis. Recently, ARBs were demonstrated to be involved in the regulation of cell cycle. Telmisartan, an AT1R blocker, blocked the S to G2 cell cycle transition by down-regulating cyclin A2 and cyclin-dependent kinase 2 (CKD2) [144]. Another study suggested that telmisartan induced cell-cycle arrest in G0-phase by reducing cyclin D1 and cyclin E [145]. Moreover, it also diminished the phosphorylation of ErbB2, ErbB3 (also known as HER3), and EGFR, as well as the expression levels of thrombospondin-1, thus inhibiting cell invasion and metastasis in ESCC cell lines [144,145]. The inhibitory effects of telmisartan on tumor development have also been identified in ESCC/EAC xenograft mouse models [144,145]. Telmisartan treatment reduced tumor volumes in ESCC/EAC xenograft mouse models by regulating the AMP-activated protein kinase (AMPK)/mammalian target of rapamycin (mTOR) transduction pathway and inducing cell cycle arrest [144,145]. Indeed, AT1R plays a critical role in the regulation of tumor progression. Given that BE with dysplasia is a primary precancerous lesion of EAC and RAS is correlated with BE dysplasia, a Swedish team further conducted a prospective randomized trial of 18 patients with low-grade dysplasia (LGD) in BE. This study explored changes of protein expression after a three-week period of ACEI/AT1R antagonist treatment using two-dimensional gel electrophoresis and mass spectrometry [161]. Available data showed that ACEI/AT1R antagonist treatment significantly inhibited heat shock protein 60 (HSP60) and protein disulphide isomerase A3 (PDIA3) expression and increased inorganic pyrophosphatase (PPA1) expression [161]. However, a close association between a high expression of PDIA3 and a favorable prognosis of ESCC has been demonstrated in another study [162]. The contrasting roles of PDIA3 in BE and ESCC could be explained by different histological types, disease types, and disease stages. Other underlying mechanisms warrant further exploration.

In addition to the angiotensin II/AT1R axis, another bypass, the angiotensin-(1-7)/mitochondrial assembly receptor (MasR) axis, is also involved in the progression of ESCC. The available evidence suggested that the angiotensin-(1-7)/MasR signaling pathway inhibited cell proliferation and angiogenesis, and those patients with a high MasR expression had better disease outcomes than those with a low MasR expression [163]. This bypass loop, along with other bypass loops, may have an influence on the action of ACEIs/ARBs and account for the different effects of ACEIs/ARBs on cancer development [69].

Caution also needs to be exercised when interpreting roles of angiotensin II receptors in esophageal cancer, since some results were obtained in a single center and a small sample. Validating these results in larger and multi-center studies has become increasingly important. Additionally, one study has found 36 upregulated miRNAs and 23 downregulated miRNAs after AT1R antagonist treatment, in comparison with controls [145]. Further studies are warranted to uncover interactions between miRNAs and angiotensin II receptors, aiming to reveal the different roles of epigenetic modifications in angiotensin II receptors and associated signaling pathways.

#### 3.3.3. Estrogen-Related GPCRs

There is an evident male predominance of EAC and ESCC, with a global overall male-to-female incidence ratio of 4.4 and 2.7, respectively [14]. A large meta-analysis further described sexual dimorphism in GERD. A pooled male-to-female incidence ratio of BE, erosive reflux disease (ERD), and nonerosive reflux disease (NERD) is 1.96, 1.57, and 0.72, respectively [164]. In comparison with males, females with esophageal cancer exhibited improved survival rates [165]. Wang et al. [166] demonstrated that serum estradiol levels were reduced in both female and male patients with ESCC than healthy controls. These epidemiologic and clinical studies suggested potential contributions of estrogen and estrogen-related receptors in esophageal diseases.

In 2018, one study investigated GPER expression in esophageal cancer. It suggested that the overexpression of GPER was associated with poor overall survival and progression free survival in patients with ESCC [146]. Moreover, patients with an advanced cancer stage presented high expression levels of GPER and Beclin-1. Activation of GPER promoted cell proliferation and increased the expression levels of Beclin-1, MAPK, and p38 MAPK. It is noteworthy that the GPER-mediated overexpression of Beclin-1 was markedly reversed by the p38 MAPK inhibitor. This finding indicated that GPER may enhance Beclin-1 expression via the p38 MAPK signal and promote ESCC progression [146]. As for the roles of GPER in esophageal carcinoma, there is still a lack of knowledge on it. However, in the study of esophageal cancer, more studies focused on classic ERα and ERβ. Wang et al. [167] claimed that activation of ERα by estradiol suppressed the proliferation and migration of esophageal cancer cell lines (EC109 cells) by inducting ERS-mediated apoptosis. A significant correlation between a higher expression of ERβ and poorer disease outcomes has also been identified [166]. Most importantly, tissue ERβ expression represented a stepwise increase in the progression from basal cell hyperplasia to dysplasia, indicating a critical role of ERβ in the development of ESCC [166]. Moreover, in mouse models of esophagitis, 17β-estradiol was claimed to be protective in esophageal epithelial injury. It downregulated the levels of TNF-α and exogenous nitric oxide (NO) and inhibited the inflammatory reaction by activation of ERα and ERβ [168].

Studies on GPER in esophageal cancer are still in the initial stages, but rapid advancements have been made in CRC and breast cancer [15,169]. Investigating the overlapping signaling pathways and genetic profiles may contribute to elucidating the functions of GPER in esophageal cancer. Moreover, estrogen-related GPCRs are expressed in various cell types, identifying the roles of estrogen-related GPCRs in different cell types is also required. Furthermore, the available evidence suggested that the stimulation of ERα and ERβ by estrogen substantially improved esophageal epithelial barrier function by increasing the expression levels of tight junction proteins and remodeling cellular architecture. Esophageal cancer is characterized by esophageal epithelial barrier dysfunction, yet the protective role of GPER, ERα, and ERβ regarding the esophageal barrier in esophageal cancer remains to be explained. Most importantly, tissue ERβ expression represented a stepwise increase in the progression of ESCC. However, whether GPER represented a stepwise increase in this progress is unresolved. Further studies are needed to clarify this issue.

#### 3.3.4. Other GPCRs

Protease-activated receptor 1 (PAR1) and PAR2 were highly expressed in esophageal cancer tissues in comparison with paired noncancerous tissues [147,170]. Patients with a high expression of PAR1 had an advanced tumor node metastasis (TNM) stage, as compared to patients with a low expression. PAR1 may enhance cell proliferation via reducing apoptosis [147]. PAR2 was also claimed to be a tumor promoter. It boosted cell invasion and migration by activating the MEK/ERK and PI3K/Akt signaling pathways and increasing the expression of MMP-9 and transmembrane 4 superfamily 3 (TM4SF3) [148]. Moreover, it was also implicated in cell cycle regulation. Down-regulation of PAR2 induced cell-cycle arrest in the S phase, thereby attenuating cell proliferation [149]. In contrast to PAR1 and PAR2, PAR4 was regarded as a tumor suppressor in esophageal cancer [150]. A lower expression level of PAR4 was found in esophageal carcinoma when compared with adjacent tissues. The activation of PAR4 lead to an increase in p16 and a decrease in DNA methyltransferase 1 (DNMT1) and histone deacetylase 2 (HDAC2), thus repressing cell proliferation and inducing apoptosis [150]. Other GPCRs including GPR120 and CCK2R were also involved in the development and progression of esophageal cancer. A high expression level of GPR120 was associated with an advanced cancer stage. GPR120 exerted tumor-promoting effects by promoting EMT and activating the PI3K/Akt and NF-κB signaling pathways [151]. Similarly, gastrin may promote cell proliferation via a CCK2R-mediated mechanism in Barrett’s carcinogenesis [152,153]. COX-2, the identified contributor in cancer progression, was increased in the gastrin/CCK2R axis [153]. It is noteworthy that a high-fat diet boosted the progression of BE to EAC in mice, and FFARs were differentially expressed in GERD [171,172]. Nevertheless, few studies have investigated the roles of FFARs in EAC or ESCC. Obviously, this is a promising area for future research.

## 4. Targeting GPCRs in GI Cancer Therapy

As the largest family of membrane receptors, GPCRs are involved in numerous cellular processes including cell proliferation, migration, and invasion, as well as angiogenesis, cell death, and cell survival, thus receiving increased attention in cancer therapy [5]. In 1942, the first drug targeting AT1R was approved by the FDA [9]. By July 2017, drugs targeting GPCRs accounted for 34% of the FDA-approved drugs (475 in total) and 60% of the world’s top 20 bestselling drugs, generating over USD 108 billion annually in sales [5,21,23].

In recent years, anti-cancer drug discovery gained considerable momentum due to the successful development of biased agonism, the identification of allosteric modulation, and the characterization of an ECD binding pocket and an intracellular pocket. Biased ligands stabilize GPCRs in different conformational states, thus stimulating highly specific signaling pathways [173]. Some β-arrestin-biased ligands preferentially activate β-arrestins and mediate cellular processes that are different from G protein-mediated ones. The AngII/AT1R axis is perhaps the perfect example of biased signaling. One β-arrestin-biased peptide (TRV120027) binds competitively at AT1R and antagonizes G protein-mediated signaling, thereby attenuating AT1R-induced side effects [173,174]. In addition, in the field of cancer research, β-arrestin-biased agonism was also claimed to mediate insulin-like growth factor 1 receptor (IGF-1R) degradation and cell sensitivity to the anti-IGF-1R antibody, providing a new direction for cancer therapy [175]. Allosteric modulation further regulates the structures and functions of GPCRs, therefore altering the binding affinity of GPCRs and permitting higher selectivity [176]. Identification of the ECD binding pocket and intracellular pocket can further assist researchers to design highly effective therapeutics with less side effects. A great number of studies have confirmed significant contributions of various GPCRs in CRC, gastric cancer, and esophageal cancer, which has made them become potential targets for cancer treatment (Table 4).

The SphK/S1P/S1PR signaling pathway regulates GI cancer progression through activating diverse signal molecules including β-catenin, EGF, COX-2, HER2, and E-cadherin [51,88,106,140]. Each of the S1PRs exert distinct effects in different TMEs, thus playing multifaceted roles in cancer progression and the therapeutic response. Hence, S1PRs hold significant promise in the treatment of GI cancer. Fingolimod (FTY720), an agonist for S1PRs (S1PR1, S1PR3, S1PR4, and S1PR5), has been approved for multiple sclerosis (MS) by the FDA, showing anti-tumor effects in CRC mouse models [30]. Ongoing clinical trials (TOUCHSTONE study) also claimed that ozanimod, a highly selective agonist for S1PR1 and S1PR5, showed favorable therapeutic effectiveness for ulcerative colitis (NCT01647516, NCT02531126) [177,178]. It is well established that patients with long-standing ulcerative colitis can develop CRC [81]. Whether ozanimod works on CRC remains to be elucidated. Other S1PR modulators such as siponimod (S1PR1 and S1PR5 agonist), ponesimod (S1PR1 agonist), etrasimod (S1PR1, S1PR4, and S1PR5 agonist), and amiselimod (S1PR1 antagonist) for immune diseases are in clinical trials [54]. In the field of cancer treatment, two promising drugs targeting the SphK/S1P/S1PR signal have been evaluated in various cancers. ABC294640, an SphK2-selective inhibitor, is currently in clinical trials for advanced cholangiocarcinoma and prostate cancer (NCT03414489, NCT04207255). Sonepcizumab (LT1009), a monoclonal anti-S1P antibody, has been evaluated in patients with refractory renal cell carcinoma (NCT01762033). As a result, this study has been terminated due to a lack of efficacy. Although there have been few clinical trials investigating the efficacy of S1PR modulators in patients with GI cancer, S1PRs indeed presented as promising targets in cancer treatment. The recent discovery of small molecule modulators of S1PRs may provide a new direction for GI cancer treatment.

As key components of RAS, many studies have clarified the roles of AT1R and AT2R in tumorigenesis. AT1R and AT2R are undoubtedly promising therapeutic targets as they regulate angiogenesis, immune responses, cell cycle, and EMT, as well as modulate the expansion of CAFs and CSCs [9,11,37,144]. As it is known to all, ARBs and ACEIs are classic antihypertensive drugs in wide use. The discovery of losartan, the first approved drug targeting AT1R (AT1R antagonist), represented a significant milestone in disease therapy. Many clinical trials have suggested them as effective adjunctive therapy for cancer treatment. A phase II clinical trial evaluated the efficacy of losartan in combination with FOLFIRINOX and chemoradiotherapy for patients with locally advanced pancreatic cancer. The available results indicated that this therapeutic strategy is effective, as it provided a downstaging of cancer [179]. In gastric cancer treatment, a retrospective study demonstrated that a combination with ACEIs/ARBs had a survival advantage when compared with platinum-based chemotherapy alone [180]. It should be noted that most positive results came from in vitro and in vivo studies, hence prospective clinical trials are needed to elucidate the efficacy of ARBs in the treatment of GI cancer. Moreover, the systemic adverse effects of ARBs such as hypotension might limit the clinical application in cancer treatment, especially for normotensive subjects. Therefore, developing ARBs that specifically accumulate and act in tumors holds the key to minimizing systemic side effects. An American research team recently designed pH-sensitive polyacetal-based polymers, which are degraded in an acidic environment (the pH value in TME is 6.7–7.2) [37]. Chauhan et al. [37] chemically linked ARBs to these pH-sensitive polymers; as a result, the ARB nanoconjugates delivered and released ARBs in the TME, and then exerted anti-tumor effects in tumors. Additionally, peptide-drug conjugates and other drug delivery materials such as liposomes, temperature-sensitive micelles, and redox-sensitive nanoparticles also opened new possibilities for cancer treatment. Furthermore, accumulated data have indicated the great potential of ARBs in reprogramming the TME. A combination of ARBs and immune checkpoint inhibitors promoted the generation of an immunosupportive TME, thus rendering tumors more sensitive to immunotherapies [37,181]. Further efforts should be made to unveil changes of immune cells and drug distribution after combination therapy. Exploring the efficacy of combined treatment of ARBs and other immune checkpoint inhibitors such as cytotoxic T-lymphocyte antigen-4 (CTLA-4) in different types of cancer is also warranted.

Estrogen-related receptors, including ERα, ERβ, and GPER, are the most studied pharmacological targets in breast cancer and endometrial cancer. Recent studies have also demonstrated critical roles of these receptors in the development of GI cancer. The associations between estrogen-related receptors and angiogenesis, DNA repair, apoptosis, ER stress, epithelial barrier, and gut microbiota have made them become excellent therapeutic targets for cancer treatment [12,81,182,183]. ERα, ERβ, and GPER were claimed to be tumor promotors or tumor suppressors in vivo and in vitro studies. Some clinical studies also observed sexual dimorphism in GI cancer [12,13,14]. It should also be stressed that the existing clinical evidence was largely obtained from retrospective studies, highlighting the need of conducting prospective validation studies in the future. A randomized, double-blind, and placebo-controlled study investigated the efficacy of eviendep (an ERβ agonist) in patients with recurrent colonic adenocarcinoma (NCT01402648). Unfortunately, results have not been published. LNS8801, an orally bioavailable, selective agonist of GPER, shows anti-tumor effects as monotherapy and combination therapy in breast cancer treatment. Recently, a phase I, open-label, multi-center study further characterized the anti-tumor effects of LNS8801 alone and in combination with immunotherapy (pembrolizumab, an anti-PD-1 antibody) in patients with solid tumors or lymphoma (NCT04130516). This study is in progress. In recent years, selective estrogen receptor degraders (SERDs) have received widespread attention in the field of breast cancer. SERDs show superiority over selective estrogen receptor modulators (SERMs) in inducing the degradation of estrogen receptors [184]. Therefore, SERDs produce more durable responses and improve disease outcomes, making it an appealing target for other kinds of cancers.

As for other therapeutic targets, plerixafor (AMD3100), a CXCR4 small molecule inhibitor (SMI), was evaluated in an open label, phase I study for advanced colorectal adenocarcinomas, pancreatic cancer, and ovarian cancer (NCT02179970). However, due to the short duration of treatment, no significant clinical response has been found [185]. Another phase IIa, open-label clinical trial (NCT02826486) assessed the therapeutic efficacy of Motixafortide (BL-8040, a CXCR4 antagonist) in metastatic pancreatic adenocarcinoma and showed favorable results regarding objective response rate, overall survival, and disease control rate [186]. SX-682, an SMI of CXCR1/CXCR2, is currently in a phase Ib/II trial with metastatic CRC (NCT04599140). Moreover, GPCR-mediated Wnt signaling is also a promising therapeutic target. Ipafricept (OMP-54F28), a recombinant fusion protein, fuses the ECD of a frizzled receptor to an IgG1 Fc fragment, thus blocking the Wnt signaling [187]. Several clinical trials evaluated the efficacy of ipafriceptin in a solid tumor (NCT01608867 and NCT02069145).

In recent years, considerable progress has been made in GPCR-targeted therapy. Methodological and technical improvements provide an impetus for uncovering the structures and functions of GPCRs. The application of structure-based design further accelerated the progress of drug discovery. As a result, GPCR-targeted peptide drugs, GPCR-targeted SMIs, and antibody-based GPCR therapeutics were approved for clinical use and innovated cancer therapy [5,19]. Emerging information on the ICD and associated signaling proteins may provide a new strategy for drug discovery. Although many GPCR-targeted drugs have been clinically prescribed for patients, especially those with central nervous system diseases or gynecological cancer, GPCR-targeted therapeutics for GI cancer are still lacking. Numerous results have been obtained from animal experiments and in vitro studies. Therefore, further efforts are needed to facilitate GPCR translational research in GI cancer. Furthermore, GI cancers are characterized by heterogeneity, with marked differences in genomic and phenotypic features. Given that genomic heterogeneity was regarded as a prominent contributor to therapeutic failure, the application of pharmacogenomics in cancer therapy is much needed [188]. The selection of targeted therapeutics for ideal individuals indeed holds the key to precision medicine in cancer treatment.

## 5. Conclusions

GI cancers are common and devastating diseases with high incidence and prevalence rates worldwide. Despite the significant advances that have been made in GI cancer treatment, the mortality is still extremely high. Recent progress in elucidating the structures and functions of GPCRs has provided new insights into the pathogenesis and treatment of GI cancers. GPCRs play a critical role in a variety of GI cancer associated-cellular processes, including cell proliferation, migration, and invasion, as well as angiogenesis, cell death, and cell survival. Significant correlations between GPCRs and treatment response have allowed GPCRs to become important therapeutic targets. Methodological and technical improvements significantly accelerated the progress of drug discovery, and many GPCR-targeted peptide drugs and SMIs have entered clinical practice. However, GPCR-targeted therapeutics for GI cancer are still in the initial stages; therefore, translational research on GPCR-targeted drug discovery is currently an unmet need. Furthermore, exploring the roles of GPCRs in *H pylori* infection and epithelial barrier dysfunction, and investigating the interplay between GPCRs and gut microbiota and epigenetic modifications are also warranted. Moreover, the existing positive results were largely obtained from in vitro and in vivo studies, highlighting the need to conduct prospective clinical trials in the future. Precision medicine is a major area of interest within the field of healthcare. To accelerate the progress of precision medicine, the application of pharmacogenomics in cancer therapy is also needed. Undoubtedly, GPCRs are prospective therapeutic targets for GI cancer. Combined efforts should be made to further facilitate the GPCR-targeted drug discovery.

## Figures and Tables

**Table 1:** Roles of G protein-coupled receptors in colorectal cancer.

GPCRs	Roles	References
**Sphingosine 1-phosphate receptors**		
S1PR1	Aggravates intestinal inflammation and promotes colitis-associated colorectal tumorigenesis	[30]
S1PR2	Inhibits tumorigenesis and reverses 5-FU chemoresistance	[31,32]
S1PR3	Promotes tumorigenesis	[33]
S1PR4	Limits CD8^+^ T cell expansion, inhibits cancer proliferation, and reduces chemotherapy success	[34]
S1PR5	Promotes CRC growth, migration, and invasion	[35]
**Angiotensin II receptors**		
AT1R	Promotes tumorigenesis and regulates cancer immunotherapy	[11,36,37,38]
AT2R	Increases the expression levels of E-cadherin	[11]
**Estrogen-related GPCRs**		
GPER	Shows bidirectional effects on tumorigenesis	[15,39,40,41]
**Other GPCRs**		
FFAR2	Shows bidirectional effects on tumorigenesis	[42,43,44]
GPR109A	Inhibits colon inflammation and tumorigenesis	
FFAR3	Enhances cell proliferation and inhibits apoptosis	[45]
CCK2R	Promotes tumorigenesis	[46,47]
GPR56	Enhances EMT and promotes chemoresistance	[48,49]

S1PR: sphingosine 1-phosphate receptor; CRC: colorectal cancer; AT1R: angiotensin II receptor type 1; AT2R: angiotensin II receptor type 2; GPCRs: G protein-coupled receptors; GPER: G protein-coupled estrogen receptor; FFAR: free fatty acid receptor; CCK2R: cholecystokinin 2 receptor; EMT: epithelial-to-mesenchymal transition.

**Table 2:** Roles of G protein-coupled receptors in gastric cancer.

GPCRs	Roles	References
**Sphingosine 1-phosphate receptors**		
S1PR1	Promotes cell proliferation and invasion	[86]
S1PR2	Shows bidirectional effects on cell migration	[87,88]
S1PR3	Promotes cell migration	[87]
**Angiotensin II receptors**		
AT1R	Promotes tumorigenesis and aggravates gastric inflammation	[89,90,91,92,93,94,95]
AT2R	Promotes tumorigenesis and aggravates gastric inflammation	[89,91]
**Estrogen-related GPCRs**		
GPER	Shows bidirectional effects on tumorigenesis and regulates chemoresistance	[16,96,97]
**Other GPCRs**		
CXCR1	Promotes cell migration and invasion	[98]
CXCR2	Promotes cell migration and invasion	[98]
CXCR3	Improves therapeutic efficacy of PD-L1/PD-1	[99]
CXCR4	Promotes metastasis and increases 5-FU chemosensitivity	[100,101]
CXCR6	Promotes cell proliferation and migration	[102]
CXCR7	Promotes cell proliferation and migration	[103]
CCK2R	Promotes tumorigenesis	[104]

S1PR: sphingosine 1-phosphate receptor; AT1R: angiotensin II receptor type 1; AT2R: angiotensin II receptor type 2; GPCRs: G protein-coupled receptors; GPER: G protein-coupled estrogen receptor; CXCR: C-X-C chemokine receptor; PD-L1/PD-1: programmed death-ligand 1/programmed cell death protein 1; CCK2R: cholecystokinin 2 receptor.

**Table 3:** Roles of G protein-coupled receptors in esophageal cancer.

GPCRs	Roles	References
**Sphingosine 1-phosphate receptors**		
S1PR1	Promotes cell proliferation and inhibits apoptosis	[139]
S1PR2	Promotes tumorigenesis	[140,141]
S1PR3	Promotes Akt phosphorylation, and regulates radiation resistance	[142]
S1PR5	Inhibits cell proliferation and migration	[58]
**Angiotensin II receptors**		
AT1R	Promotes cell proliferation and angiogenesis	[143,144,145]
**Estrogen-related GPCRs**		
GPER	Promotes cell proliferation	[146]
**Other GPCRs**		
PAR1	Promotes cell proliferation	[147]
PAR2	Promotes cell invasion and migration	[148,149]
PAR4	Inhibits cell proliferation	[150]
GPR120	Promotes EMT and cancer progression	[151]
CCK2R	Promotes cell proliferation	[152,153]

S1PR: sphingosine 1-phosphate receptor; AT1R: angiotensin II receptor type 1; GPCRs: G protein-coupled receptors; GPER: G protein-coupled estrogen receptor; PAR1: protease-activated receptor 1; PAR2: protease-activated receptor 2; PAR4: protease-activated receptor 4; EMT: epithelial-to-mesenchymal transition; CCK2R: cholecystokinin 2 receptor.

**Table 4:** List of drug candidates targeting GPCRs.

Compound	Targets	Indications	Clinical Phase
Fingolimod	S1PR1, S1PR3, S1PR4, and S1PR5 agonist	Multiple sclerosis	Approved
Ozanimod	S1PR1 and S1PR5 agonist	Ulcerative colitis	Phase II
Siponimod	S1PR1 and S1PR5 agonist	Multiple sclerosis	Approved
Ponesimod	S1PR1 agonist	Multiple sclerosis	Approved
Etrasimod	S1PR1, S1PR4, and S1PR5 agonist	Ulcerative colitis and Crohn’s disease	Phase II and Phase III
Amiselimod	S1PR1 antagonist	Ulcerative colitis	Phase II
LNS8801	GPER agonist	Solid tumor and adult lymphoma	Phase I and Phase II
Plerixafor	CXCR4 SMI	Advanced colorectal adenocarcinomas, pancreatic cancer, and ovarian cancer	Phase I
Motixafortide	CXCR4 antagonist	Metastatic pancreatic adenocarcinoma	Phase II
SX-682	CXCR1 and CXCR2 SMI	Metastatic colon adenocarcinoma	Phase I and Phase II

GPCRs: G protein-coupled receptors; S1PR: sphingosine 1-phosphate receptor; GPER: G protein-coupled estrogen receptor; CXCR: C-X-C chemokine receptor; SMI: small molecule inhibitor.
